# Patterns of Focal- and Large-Scale Synchronization in Cognitive Control and Inhibition: A Review

**DOI:** 10.3389/fnhum.2020.00196

**Published:** 2020-06-25

**Authors:** Carolina Beppi, Ines R. Violante, Adam Hampshire, Nir Grossman, Stefano Sandrone

**Affiliations:** ^1^Neuroscience Center Zürich (ZNZ), University of Zürich (UZH) and Swiss Federal Institute of Technology in Zürich (ETH), Zurich, Switzerland; ^2^Department of Neurology, University Hospital Zürich, University of Zürich, Zurich, Switzerland; ^3^Computational, Cognitive and Clinical Neuroscience Laboratory (C3NL), Department of Brain Sciences, Imperial College London, London, United Kingdom; ^4^School of Psychology, Faculty of Health and Medical Sciences, University of Surrey, Guildford, United Kingdom; ^5^Department of Brain Sciences, Imperial College London, London, United Kingdom

**Keywords:** neural oscillations, de(synchronization), stop-signal task, Go/No-go task, response inhibition, cognitive inhibition, interference suppression, rule inhibition

## Abstract

Neural synchronization patterns are involved in several complex cognitive functions and constitute a growing trend in neuroscience research. While synchrony patterns in working memory have been extensively discussed, a complete understanding of their role in cognitive control and inhibition is still elusive. Here, we provide an up-to-date review on synchronization patterns underlying behavioral inhibition, extrapolating common grounds, and dissociating features with other inhibitory functions. Moreover, we suggest a schematic conceptual framework and highlight existing gaps in the literature, current methodological challenges, and compelling research questions for future studies.

## Introduction

Investigating the relationship between cognitive function and underlying cerebral activity has been, and still is, one of the greatest neuroscientific challenges. Functional magnetic resonance imaging (fMRI) is a leading imaging method for quantifying and mapping the geographical distribution of metabolic changes associated with brain activity, while resting (Riedl et al., 2016) or actively processing information (Chen and Glover, 2015). Electroencephalography (EEG) is a well-established electrophysiological technique providing a temporally accurate recording of postsynaptic superficial brain activity (Burle et al., 2015), safely and non-invasively (Cohen, 2017), at rest or during task performance (Zani and Proverbio, 2003). Together with magneto-electroencephalography (MEG), EEG has extensively contributed to the understanding of how the brain’s oscillations at different frequencies relate to specific mental states and processes (Benedek et al., 2014). Moreover, it permits to measure local alterations in amplitude, phase, and synchrony, and to explore spatial and temporal distributions associated with specific cognitive functions (Perfetti et al., 2011; Groppe et al., 2013; Roux and Uhlhaas, 2014), such as attention and memory. This article will review the current knowledge of the patterns of focal and large-scale coordination supporting cognitive control and inhibition.

### The Importance of Large-Scale Synchronization in Complex Cognitive Functions

Increased EEG/MEG amplitude, power and event-related synchronization (ERS), or desynchronization (ERD), within local circuits and specific frequencies support distinct cognitive processes, including sensory processing and memory (reviewed in Uhlhaas et al., 2008; Roohi-Azizi et al., 2017). However, functional connectivity reports suggest that more complex cognitive tasks, involving a dynamic combination of cognitive processes, require a fast and adjustable information exchange between brain circuits of large scale (Hampshire et al., 2012). More emphasis is thus now given to investigating coordination processes between long-range neural networks and the underlying neurobiological mechanisms during more demanding cognitive functions (Fries, 2005; Fell and Axmacher, 2011; Kazanovich, 2019; Wang et al., 2019).

Phase-synchronization processes ease the information exchange within distributed brain networks, increasing network efficiency, and facilitating synaptic plasticity (Varela et al., 2001; Fries, 2005, 2015; Womelsdorf et al., 2007; Deco et al., 2011; Fell and Axmacher, 2011; Parkin et al., 2015; Constantinidis and Klingberg, 2016; Violante et al., 2017). The importance of intact long-range synchronization dynamics is evident in clinical contexts, where cognitively impaired Alzheimer’s patients display significantly decreased phase-coordination between most cortical regions in the delta band, relative to controls (Hata et al., 2016). This calls for the need for further investigations and the development of methods (e.g., Pesaran et al., 2018; Widge et al., 2019a) to study synchronization patterns between large-scale networks and the underlying synaptic mechanisms, as well as their alterations in different neuropsychiatric diseases.

The study of large-scale synchronization implies recording neural activity contemporaneously from distributed brain locations before assessing whether the activity at different loci alters in a synchronous fashion (Nowak et al., 2017). Activity within single voxels or region-of-interests is tracked measuring the correlation across them over time series (Harris and Gordon, 2015). Long-range phase-synchronization dynamics between large-scale circuits can be explored within the same, or between a broad span of, different frequencies in EEG/MEG. Phase-coordination in different frequencies is a type of cross-frequency coupling (CFC), called “cross-frequency phase-phase coupling” (Palva et al., 2005). Phase-amplitude coupling is another type of CFC, which describes the synchronization of the phase of a low-frequency rhythm to the amplitude/power of a higher-frequency rhythm (Canolty and Knight, 2010).

Long-range phase-coordination between distributed frontal/executive and sensory networks is associated to increased cognitive demand, as a result of increased sensory-processing (Crespo-Garcia et al., 2013), manipulation of sensory information in working memory (Sauseng et al., 2005), as well as memory encoding and retrieval (Schack and Klimesch, 2002; Sauseng et al., 2004; Schack et al., 2005). The contribution of different network components in a given task is dynamic in time and extent and depends on the specific cognitive requirement.

Cross-frequency phase-phase/amplitude coupling dynamics have been described in working memory processes. However, it is yet to be established how they apply to behavioral inhibition, a complex function that relies on a combination of cognitive processes, including attention, working memory, action selection (Hampshire et al., 2007; Stokes et al., 2013; Provenza et al., 2019; Widge et al., 2019a), and that is likewise distributed across brain networks (Erika-Florence et al., 2014; Hampshire and Sharp, 2015). Existing knowledge and evidence in this regard will be reviewed and elucidated in the following sections.

## Focal and Distributed Patterns of Neural Synchrony in Behavioral Inhibition

### Control and Inhibition—Brief Conceptual Definitions

**Figure 1:**
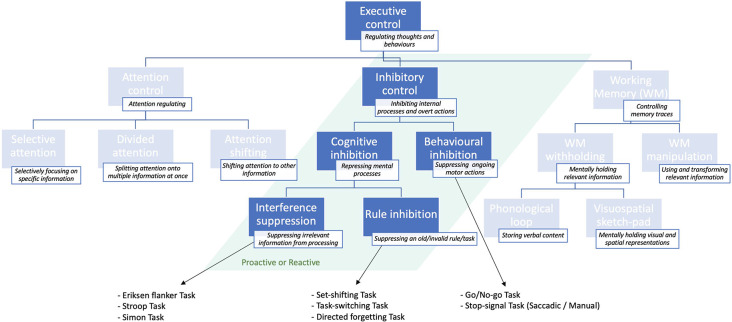
Schematic illustration of the inhibitory sub-functions of executive control presented in descending order of specificity: from more general (top) to more specific (bottom) and classical tasks that are used to study them. For a more exhaustive overview of executive control sub-functions, see Jones et al. (2016).

Executive control is a major cognitive function comprising several sub-functions (Jewsbury et al., 2016; Purpura et al., 2017), including attentional control and working memory. But it encompasses also inhibitory control or inhibition (Jones et al., 2016), which regulates flexible and adaptive overt responses as well as purpose-directed mental processes (Stuphorn and Emeric, 2012). The ability to inhibit an internal process, or to interfere with external information is generally referred to as inhibitory control or, simply, inhibition (Xie et al., 2017). The latter is, in turn, distinguished between behavioral or response inhibition, which refers to the process of suppressing an ongoing motor action, whenever necessary (e.g., to implement an alternative response; Aron, 2007). The most established paradigms used to study behavioral inhibition are summarized in Box 1. Cognitive inhibition (Bari and Robbins, 2013), instead, involves the blockade of a mental process, such as selective attention or memory retrieval (MacLeod, 2007), either intentionally or unconsciously (Harnishfeger, 1995). A schematic representation of the different inhibitory subfunctions is shown in Figure 1.

Box 1 **Classical response inhibition tasks**

A variety of behavioral tasks have been developed and used to study the neural underpinnings of behavioral inhibition, among which the most established constitute the Go/No-go task (GNGT; e.g., Luijten et al., 2011; Uzefovsky et al., 2016) and stop-signal task (SST; e.g., Jahfari et al., 2010; Leunissen et al., 2016). Alternative paradigms include the antisaccade (e.g., Tervo-Clemmens et al., 2017; Fernandez-Ruiz et al., 2018) and the delayed-gratification tasks (e.g., Jiang et al., 2018).

In the GNGT, the subject is presented with a series of different stimuli (e.g., arrows on a screen) and must respond to those defined as target (e.g., left-pointing arrows) by taking a given action (e.g., button-press) as fast as possible. Upon occurrence of any non-target stimuli (e.g., right-pointing arrows) the participant must instead suppress the response and not press. The task can be implemented using different stimuli, sensory modalities and response effectors. The GNGT performance can be quantified in terms of reaction time to target stimuli (aka “go-trials”) and frequency of correct/incorrect presses and correct/incorrect suppressions, which would define the accuracy.

In the SST, the subject must respond to different stimuli (e.g., left/right-pointing arrows) by selecting the corresponding response option (i.e., left/right button-press based on arrow orientation) and inhibit the response whenever an additional infrequent stimulus (e.g., audio-tone), namely the stop-signal (SS), is presented (Ko et al., 2016). Performance on the SST can be quantitatively modeled as a horse-race model (Logan et al., 1984), where a competition between the “go” and “stop” processes determines behavior, producing an estimation of the SS reaction time (SSRT), which is the time necessary for suppressing the motor response (Band et al., 2003; Boucher et al., 2007). The latter largely depends on the type of effectors chosen for selecting the response, with estimations averaging from 130 ms in the saccade SST (Hanes and Carpenter, 1999) to 250 ms in the manual SST (Boucher et al., 2007). The delay of the SS relative to the antecedent (go) stimulus determines the stop success probability (Logan et al., 1984). In the classical SST, the SS-latency is defined through a staircase design, which enables to adjust the paradigm to the individual performance, narrowing it on to the 50% success probability of making the stop (Erika-Florence et al., 2014). Other relevant outcome measures include direction errors, percentage of successful stops, and RT in go-trials (Stuphorn and Emeric, 2012).

Cognitive inhibition can be further distinguished into sub-types. The act of preventing irrelevant sensory information from undergoing further processing in working memory (Wilson and Kipp, 1998; Diamond et al., 2013) is known as interference suppression (Nigg, 2000), and can be assessed through the Eriksen flanker task (Eriksen and Eriksen, 1974). This task requires the subject to focus on the target letter in the center, ignoring the neighboring letters (flankers), which are either matching, neutral, or unmatching the central letter concerning a specific feature (e.g., color, shape, size). Such experiments demonstrated that people are generally slower and more inaccurate at responding in target-unmatching, relative to target-matching, trials (Eriksen, 1995). This finding is known as “stimulus-response compatibility” effect (e.g., Richez et al., 2016). Variants to the Eriksen paradigm include the Stroop task (Stroop, 1935), and the Simon task (Simon and Wolf, 1963).

The process of actively suppressing an invalid rule or “task-goal” is instead known as rule inhibition (Xie et al., 2017), and is typically studied with directed forgetting and “task-switching” or “set-shifting” paradigms (Nigg, 2000; Monsell, 2003; Koch et al., 2010). As opposed to interference suppression, rule inhibition involves a working memory component, in that subjects must actively suppress an old (invalid) task-goal or rule, while mentally maintaining and applying the new (valid) task/rule (Xie et al., 2017).

Within the scientific literature, a distinction is often made between the proactive and reactive forms of inhibitory control (Braver et al., 2008; Braver, 2012). Proactive inhibition represents an anticipatory form of selection, by which goal-relevant information is actively and continuously maintained in working memory to direct attentional, perceptual, and motor systems (Miller and Cohen, 2001). In other words, it cues attention according to the current goal, preventing interference, and thus allowing optimal performance (Stuphorn and Emeric, 2012). Reactive inhibition is, instead, a late form of control acting as a corrective mechanism that is transiently implemented when encountering an interfering event (Jacoby et al., 1999). It allows a reformulation of the goal based on episodic associations (i.e., previous experience) or/and interference demands (Stuphorn and Emeric, 2012). The proactive and reactive inhibition mechanisms complement each other in terms of advantages and disadvantages, since the first is less prone to interference, although more cognitively demanding than the second (Braver et al., 2008; Mäki-Marttunen et al., 2019). Proactive and reactive inhibitory control have been probed in both behavioral inhibition (Verbruggen and Logan, 2009; Benis et al., 2014; Castro-Meneses et al., 2015) and task-switching studies (Braver et al., 2008; Karayanidis and Jamadar, 2014).

### A Focal Brain Region Supporting Behavioral Inhibition?

The neural basis of inhibitory control functions is a research topic that has received conspicuous attention in the cognitive neurosciences in the last few decades. This focus is in part because deficits of response inhibition and cognitive flexibility characterize several neuropsychological conditions, including obsessive-compulsive disorder (OCD; McLaughlin et al., 2016), schizophrenia (Hughes et al., 2012), as well as, post-traumatic-stress-disorder (PTSD; Clausen et al., 2017), depression (Katz et al., 2010), drug-addictions (Morein-Zamir and Robbins, 2015; Wang et al., 2018) and attention-deficit-hyperactivity-disorder (ADHD; Hwang et al., 2019).

Functional neuroimaging studies have provided essential insights into the distribution of the cortical circuits underlying behavioral inhibition. The earliest works have built extensively on a modular view of the inhibitory functions, supporting that the right inferior frontal gyrus (IFG) and the anterior insula (AI) are brain regions specifically devoted to response inhibition (Aron et al., 2003, 2004), primarily based on the association of these areas’ activity with the successful withholding of automated (go-signal) responses in stop-signal tasks (SSTs; Rubia et al., 2001a,b). In support of this, selective disruptions of IFG activity compromise SST performance (Aron et al., 2003). Clinically, dysfunctional activations of the IFG and AI are observed in subjects with impulse-control disorders (Rubia et al., 1999, 2014; Seeley et al., 2009; Jilka et al., 2014). However, this is arguably overly simplistic, since the activation of these two regions is not specific to behavioral inhibition (Shallice et al., 2008; Hampshire et al., 2010; Sharp et al., 2010; Erika-Florence et al., 2014; Hampshire, 2015).

Saccadic and manual SST studies have demonstrated that, additionally to the IFG and AI, a wide circuit of frontoparietal structures, including the supplementary eye fields, the supplementary and pre-supplementary motor cortices (Curtis et al., 2005; Aron and Poldrack, 2006; Li et al., 2006; Aron et al., 2007) and the intraparietal sulcus (Osada et al., 2019), support response inhibition in collaboration with the limbic basal ganglia. Notably, the striatum (Zandbelt and Vink, 2010; Mallet et al., 2016) and the subthalamic nucleus (Brittain et al., 2012; Alegre et al., 2013).

Functional connectivity analyses have probed the co-activation of the IFC and AI with spatially distributed subcortical and frontoparietal structures (Dosenbach et al., 2008; Mostofsky and Simmonds, 2008; Duann et al., 2009; Hampshire et al., 2012; Zhang and Li, 2012; Cai et al., 2019) that compose the Multiple Demand Cortex (MDC; Hampshire and Sharp, 2015), whose contribution in behavioral inhibition differs in the extent, depending on the sensory modality and cognitive demands (Erika-Florence et al., 2014). Relative to the AI, the IFG is more involved in implementing inhibitory control and more strongly connected to the dorsomedial PFC and lateral frontoparietal cortices (Cai et al., 2014). The AI, instead, predominantly deals with the detection of salient inhibitory cues and shows a stronger intrinsic functional connectivity with the ACC (Cai et al., 2017) and the STN (Cai et al., 2019). The latter contributes to proactive and reactive inhibitory processes through distinct EEG spectral patterns (Benis et al., 2014).

### Distributed Synchronization Patterns in Cognitive Control and Response Inhibition

Response-inhibition is produced by inhibitory processes that are ubiquitous in the human brain, namely lateral inhibition and top-down potentiation (Desimone and Duncan, 1995; Chelazzi et al., 1998). These are enacted at the level of both local neuronal populations and long-range networks (MacLeod, 2007; Hampshire and Sharp, 2015). Motor responses in the SST are modulated and adjusted online by top-down (or feedforward) control signals originating from the MDC (Hampshire and Sharp, 2015) and by bottom-up (or feedback) processes of lateral-inhibition, occurring at the level of local sensorimotor neuronal populations (Boucher et al., 2007; Schall and Godlove, 2012) that support competing motor programs (Munakata et al., 2011).

While button presses to go-signals are automated responses produced *via* direct sensorimotor mappings, blocking a routine response is a non-automated process that requires the additional intervention of higher-order frontoparietal circuits (Hampshire and Sharp, 2015). The detection of a stop-signal results in the activation of the MDC (Hampshire et al., 2007; Stokes et al., 2013; Erika-Florence et al., 2014) and the sensorimotor cortex (Hampshire et al., 2007; Erika-Florence et al., 2014). The first would reinforce the motor program for the stop, while downregulating the sensorimotor representations *via* lateral-inhibition, decelerating the “go-response” and thus producing the stop outcome (Hampshire and Sharp, 2015). Upon training, response-withholding will eventually become automatic (learning), no longer requiring top-down adjustments (Erika-Florence et al., 2014; Widge et al., 2019a).

While fMRI and connectivity reports have spatially localized the neural correlates of inhibition (Curtis et al., 2005; Aron and Poldrack, 2006; Li et al., 2006; Aron et al., 2007; Zandbelt and Vink, 2010; Brittain et al., 2012; Alegre et al., 2013; Erika-Florence et al., 2014; Hampshire and Sharp, 2015; Mallet et al., 2016), electrophysiological methods can provide precise information about the development across the time of the stop-detection and response-suppression processes, exploiting their high temporal resolution. It would be especially important to explore the chronological dynamics by which the sensorimotor cortices are active, and by which the frontoparietal circuits exert their modulatory (i.e., inhibitory) action over the motor output.

Several EEG/MEG studies of response inhibition have identified characteristic event-related potential (ERP) components (i.e., N2/P2 complex) in association to the stop/no-go, but not the go, trials, in both the SST (Ramautar et al., 2006; González-Villar et al., 2016) and the Go/No-go task (GNGT; Falkenstein et al., 2002; Nieuwenhuis et al., 2004; Johnstone et al., 2007). These consist of a frontomedial negative component arising 200–300 ms after the occurrence of the stop-signal (SS), succeeded, after about 150 ms, by a frontomedial and parietomedial positive deflection. However, as we will discuss in the next paragraphs, these ERP components are not specific to behavioral inhibition, as similar patterns have also been reported in cognitive inhibition processes in Stroop (Liotti et al., 2000; Bruchmann et al., 2010), Flanker (Kopp et al., 1996) and task-switching paradigms (Karayanidis and Jamadar, 2014).

Intracranial stereoelectroencephalography (SEEG) and electrocorticography (ECoG) are invasive electrophysiological recordings of brain activity (Young et al., 2019). Although only applied to clinical populations, the direct recording from the brain tissue allows a relatively superior geographical resolution. The assessment of large-scale LFP synchronization dynamics can provide potential insights into the exact source of top-down inhibitory inputs (Widge et al., 2019a). Increased demands of top-down control, due to conflict (e.g., interference, stop-signal) detection, are indexed by cross-frequency ECoG coupling between prefrontal theta phase and the amplitude of primary motor high-frequency oscillations (Voytek et al., 2015). ECoG theta coupling accompanies information exchange from fronto medial to parietal areas upon error feedback in a Stroop-like paradigm (Smith et al., 2015), likely acting as a modulatory attentional mechanism over motor areas, augmenting the stimulus-detection probability. Theta synchrony circuits during conflict detection also convolve the dorsal cingulate cortex and subcortical structures (Provenza et al., 2019; Smith et al., 2019).

Successful response-inhibition in the GNGT provides SEEG gamma synchrony within the default mode network and the limbic system (Laviolette, 2007; Arnulfo et al., 2018). The affective attribute of response-withholding is further probed by facial electromyography, where the corrugator supercilii, a muscle closely associated with negative affect, shows higher activity in no-go, relative to go, trials (Clancy et al., 2019). This suggests that response-inhibition may negatively affect the emotional/motivational connotation of the response-associated stimulus. This is compatible with the presence of inhibitory deficits in psychiatric conditions, such as major depression and schizophrenia, involving dopaminergic dysregulations in the limbic-prefrontal (mesocortical) projection (Patel et al., 2010; Grace, 2012; Belujon and Grace, 2017). Brain stimulation (Dubreuil-Vall et al., 2019; Widge et al., 2019b) of control circuits can indeed restore both clinical symptoms and cognitive deficits in clinical populations.

### Dissociating Stop Expectancy From Response Inhibition

Investigating the functional correlates of behavioral inhibition requires the isolation of the mere behavioral act of response-withholding from its cognitive component: conflict detection or interference/stop-expectancy. In this regard, Chikazoe et al. (2009) designed a modified SST that enabled the separation of response inhibition from SS-expectancy or RT slowing, by introducing “certain go-trials” in which the SS never occurs, in addition to “uncertain go-trials” where a SS may occur, as in the classical SST. By comparing RTs to certain and uncertain go-trials, it emerged that slowing of RT to go-trials reflects the subject’s SS-expectancy and proactive inhibition, thus improving the SS reaction time (SSRT).

Stop-expectancy can be quantified trial-by-trial as stop-occurrence-probability from a dynamic Bayesian model (Yu and Cohen, 2009) and behaviorally, it correlates with RT slowing to go-signals. The spectral correlates of stop-expectancy and RT-slowing seem to be inversely related across trials (Chang et al., 2017). Stop-anticipation is accompanied by a pronounced low-theta activity in the supramarginal gyrus (SMG) and anterior SMC preceding, but not after, the occurrence of the go-signal. Slowing of RT is instead negatively associated with IFG and posterior delta-theta activity. The results suggest that stop-expectancy and response-inhibition are processed by distinct frontoparietal networks, in coordination with temporally distinguished theta contributions (Chang et al., 2017). The evidence supports earlier-discussed fMRI findings (Hu et al., 2015a,b; Manza et al., 2016) in that proactive behavioral inhibition does not map onto a specific brain region, but, instead, results from the interaction between distributed frontoparietal MDC networks (Hampshire and Sharp, 2015).

Furthermore, a simultaneous fMRI-EEG SST study (Ko et al., 2016) has shown that beta synchronization in the right medial frontal gyrus (rMFG) after the go-stimuli precedes alpha-beta suppression in the preSMA in the stop-, as opposed to go-, trials. The findings align with Chang et al. (2017) supporting that response inhibition is mediated by beta and theta activity in communication with the same MDC components. In a previous work, Swann et al. (2009) observed a stronger IFG beta (16 Hz) activity occurring 100–250 ms after the SS onset, in successful, compared to unsuccessful stop trials, accompanied by reduced synchronization in the primary motor cortex, possibly reflecting increased GABA-mediated inhibition.

Taken together, the evidence suggests that behavioral inhibition is implemented *via* IFG beta and preSMC theta activities, in communication with other frontoparietal and basal ganglia circuits, with downstream effects on the M1. Importantly, beta activity during response-inhibition processes shows opposite patterns in different MDC components, decrementing in the preSMC, but increasing in the IFG.

## Functional Dissociations Between Different Inhibitory Sub-Functions

Interference suppression and response-inhibition activate spatially overlapping, yet distinguishable, ERP correlates in combined GNGT and flanker task studies (Johnstone et al., 2009; Brydges et al., 2012, 2013; Van Velzen et al., 2014; Vuillier et al., 2016), supporting a functional dissociation between the two inhibitory subfunctions. Target-matching trials give rise to a stronger N2 component, compared to unmatching ones. However, while the P3 amplitude is higher in congruent trials involving response-suppression compared to those that do not, the N2 component seems to be unaffected (Groom and Cragg, 2015).

In a modified flanker task that allows to contemporaneously assess different inhibitory control sub-functions, Xie et al. (2017) observed that while interference suppression originates a larger frontal N2 compared to non-inhibitory trials, rule inhibition induces higher frontal P3a amplitudes, reflecting the criticality of frontal circuits in cognitive control, *via* online adjustments of stimulus-response associations. Behavioral inhibition instead shows a more substantial posterior P3b component, presumably indexing motor re-programming. Consistently, time-frequency EEG analyses confirm a fronto medial (Fz) involvement in different inhibitory sub-functions (Cavanagh and Frank, 2014; Cohen, 2014). Increased theta activity predicts slower responses to target-incongruent trials of Simon tasks or variants (Cohen and Donner, 2013; Cohen and Ridderinkhof, 2013; Clayton et al., 2015; Pastötter et al., 2010, 2013; Cohen and Cavanagh, 2011; Nigbur et al., 2011), while frontal alpha activity is instead associated with the suppression of non-relevant sensory stimuli in flanker tasks (Suzuki and Shinoda, 2015).

These findings suggest that different inhibitory sub-functions produce spatially, temporally, and quantitatively distinguishable brain activity patterns, highlighting the importance of maintaining a conceptual separation and the non-generalization of the evidence relating to any sub-functions.

## Discussion

This article reviewed the patterns of neural synchronization underlying cognitive control and behavioral inhibition. So far, electrophysiology research has demonstrated the pivotal role of focal (de)synchronization patterns within specific frequencies in different cognitive processes (reviewed in Uhlhaas et al., 2008; Roohi-Azizi et al., 2017). However, emerging evidence suggests that more complex cognitive functions, in addition to local (de)synchronizations, require the contribution and coordination of brain circuits located distally from the site of primary processing (Schack and Klimesch, 2002; Sauseng et al., 2004, 2005; Schack et al., 2005; Hampshire et al., 2012; Crespo-Garcia et al., 2013).

Phase-synchronization facilitates the communication between distributed neural circuits, by augmenting the transmission efficiency and by promoting synaptic plasticity (Fries, 2015; Parkin et al., 2015; Constantinidis and Klingberg, 2016; Violante et al., 2017). Dysfunctions in long-range synchronization are, not surprisingly, implicated in clinical neurological conditions (Hata et al., 2016), raising the necessity of new methods and research for the study of phase dynamics across distributed brain networks.

Large-scale synchronization can be observed in fMRI and EEG/MEG, where the cooperation between distributed brain regions can occur *via* phase-synchronization within the same or/and between different frequencies (Palva et al., 2005; Canolty and Knight, 2010). While phase coupling dynamics have been described in working memory (Schack and Klimesch, 2002; Sauseng et al., 2004, 2005; Schack et al., 2005; Crespo-Garcia et al., 2013), the synchronization patterns involved in inhibition, a complex function that is likewise distributed across large-scale circuits, remain elusive.

Coordination patterns between frontoparietal MDC circuits act as a modulatory top-down control mechanism over sensory areas, refining/adjusting the processing, maintenance, retrieval and manipulation of relevant information, to support cognitively demanding tasks (Sauseng et al., 2005; Hampshire et al., 2012; Crespo-Garcia et al., 2013). In behavioral inhibition, specifically, the choice of motor response (press/no-press) depends on given pre-assumptions (i.e., stimulus type), which may incur a change over time (i.e., stop signal), therefore requiring a fast adjustment and correction of the motor command (i.e., “no-press”).

The GNGT and the SST represent two well-established paradigms, by which the cognitive processes and functional neural dynamics underlying behavioral inhibition can be studied. Functional connectivity fMRI studies have been instrumental in demonstrating how response inhibition does not map onto a single dedicated brain area. Still, it is supported by the dynamic coordination between distributed frontoparietal networks, whose specific contribution (i.e., extent and spatial distribution) relates to the contextual demand (Curtis et al., 2005; Aron and Poldrack, 2006; Li et al., 2006; Aron et al., 2007; Zandbelt and Vink, 2010; Brittain et al., 2012; Hampshire et al., 2012; Alegre et al., 2013; Erika-Florence et al., 2014; Hampshire and Sharp, 2015; Mallet et al., 2016).

While fMRI has been fundamental to localize the distributed coordination dynamics during response inhibition spatially, electrophysiology focuses on the dynamic patterns of phase-synchronization over time. Notably, one would define the exact timings at which specific frontoparietal MDC components exert their modulatory action over the sensorimotor cortices. Specific EEG/MEG ERP components have been related to the stop process (Ramautar et al., 2006; González-Villar et al., 2016), although lacking specificity for a given inhibitory sub-function (Kopp et al., 1996; Liotti et al., 2000; Bruchmann et al., 2010; Karayanidis and Jamadar, 2014). Intracranial electrophysiology in clinical populations showed that theta-synchronization within fronto medial, cingulate, and parietal circuits are key components of top-down control. In addition, response-withholding has a motivational attribute and is mediated by gamma synchrony within limbic-prefrontal mesocortical projections.

Electrophysiological investigations showed that response inhibition and the “overlapping” processes of SS-expectancy, RT-slowing are accompanied by oscillatory activity that is temporally and frequency-wise distinguished for different MDC components. This highlights the necessity of experimental designs that allow their separation (e.g., Chikazoe et al., 2009; Swann et al., 2009; Ko et al., 2016; Chang et al., 2017). The evidence is still elusive due to a mismatch between the experimental designs, preventing a close comparison and direct inferences from the results.

Further research is required, and multimodal synchronized EEG-fMRI approaches (e.g., Mizuhara et al., 2005) or the more recent DSI-Hybrid-EEG-fMRI headset (e.g., Hong et al., 2018) have the potential to further elucidate patterns of focal and long-range synchronization. This will be achieved by exploiting the respective advantages of electrophysiological and hemodynamic imaging techniques in terms of temporal and spatial resolution. Neural stimulation techniques, such as transcranial alternating current stimulation (e.g., Violante et al., 2017), can manipulate the modulatory effect of prefrontal networks over sensory areas during inhibitory processes, thus allowing to draw and ascertain conclusions on the topographical and chronological distribution of the causal relations between distributed circuits.

## Author Contributions

CB and SS: major role in designing and conceptualizing the article, drafted, wrote and revised different versions of the manuscript for intellectual content. IV, AH, and NG: wrote a section of the manuscript and revised a previous version of the manuscript for intellectual content.

## Conflict of Interest

The authors declare that the research was conducted in the absence of any commercial or financial relationships that could be construed as a potential conflict of interest.
